# Adherence to the oral contraceptive pill: the roles of health literacy and knowledge

**DOI:** 10.1080/21642850.2020.1850288

**Published:** 2020-12-01

**Authors:** Caitlin Liddelow, Barbara Mullan, Mark Boyes

**Affiliations:** Health Psychology and Behavioural Medicine Research Group, School of Psychology, Curtin University, Perth, Western Australia

**Keywords:** Oral contraceptive pill, adherence, health literacy, knowledge

## Abstract

**Objective:**

The oral contraceptive pill is the most widely used method of contraception and when adhered to perfectly is 99% effective at preventing pregnancy. However, adherence to the pill is relatively low. Knowledge has shown to be important in continuation of the pill, and previous research shows the importance of health literacy in adhering to medication in chronic illnesses, but its role has yet to be explored in this behavior.

**Methods:**

This cross-sectional study examined the associations between health literacy, knowledge of the pill and adherence, as well as the predictive ability of these two variables and their interaction, in predicting adherence. Recruited through CloudResearch, 193 women (*M*_age_ = 32.63 years, *SD* = 5.98) residing in the United States completed the Health Literacy Skills Instrument – Short Form, a previously validated measure of oral contraceptive pill knowledge and the Medication Adherence Report Scale.

**Results:**

Results showed a strong positive correlation between health literacy and adherence (*r* = .76) and moderate associations between health literacy and knowledge (*r* = .42), and knowledge and adherence (*r* = .42). The final model of the hierarchical multiple regression accounted for 59.8% of variance in adherence, with health literacy (*β* = .69) and length of time taking the pill (*β* = .13) the only significant predictors of adherence.

**Conclusion:**

Family planning clinics should consider assessing the patient’s health literacy skills before prescribing the pill to ensure patients fully understand the requirements.

## Introduction

The oral contraceptive pill, otherwise known as the ‘pill’, is the most commonly used method of contraception in economically developed countries (United Nations, 2015). Between 2015–2017, approximately 80% of sexually active women in the US, between the ages of 15 and 49, indicated they had ever used the oral contraceptive pill (Daniels & Abma, 2018). The pill is highly effective at preventing pregnancy when adhered to perfectly, such that only 0.3% of women report falling pregnant in the first year (Trussell, 2011). In addition, whilst most commonly being used as a method of contraception, the pill also has many non-contraceptive related uses including reducing menstrual bleeding, menstrual cramps and menstrual-related migraines, and reducing the risk of ovarian cysts and the occurrence of acne (Arowojolu, Gallo, Lopez, & Grimes, 2012; Dayal & Barnhart, 2001; Edelman, Lew, Cwiak, Nichols, & Jensen, 2007; Vercellini et al., 2003).

Despite the numerous benefits, affordability and ease of use, adherence to the pill is still relatively low (Molloy, Graham, & McGuinness, 2012; Rosenberg, Waugh, & Burnhill, 1998). Studies have shown that perfect adherence is rare with approximately 50% of women reporting missing their pill at least once per month (Molloy et al., 2012; Rosenberg et al., 1998). It is also estimated that approximately 22% of women taking the pill miss two or more per month (Rosenberg et al., 1998). This less than perfect adherence reduces the effectiveness of the pill to only 91%, reduced from 99.7% (Trussell, 2011), and it is suggested to be the primary reason for unintended pregnancies in women using the pill (Cleland, Conde-Agudelo, Peterson, Ross, & Tsui, 2012; Cleland, Raymond, Westley, & Trussell, 2014).

Much of the research exploring adherence to the pill has focused on the correlates of non-adherence or misuse rather than understanding the factors associated with better adherence (Tomaszewski, Aronson, Kading, & Morisky, 2017). One factor associated with higher levels of adherence to the pill is knowledge of the pill, both perceived (Tomaszewski et al., 2017) and actual (Hall, Castaño, & Westhoff, 2014). Both studies showed that women who report believing they have higher knowledge of the pill or actually exhibit greater knowledge, reported higher levels of adherence and intent to continue taking the pill (Hall et al., 2014; Tomaszewski et al., 2017). Women with a higher knowledge of how the pill works, its benefits, side-effects and use were up to 6 times more likely to continue taking their pill compared to less knowledgeable women (Hall et al., 2014). Levels of knowledge were lower in younger women, perhaps suggesting that as age increases, so does knowledge of the pill.

Although not specific to the oral contraceptive pill, due to the lack of research, another factor that has shown to be associated with increased adherence to medication is health literacy. It is estimated that approximately 60% of adults have poor or inadequate health literacy skills (Australian Commission on Safety and Quality in Health Care, 2014). Research indicates that increasing levels of health literacy may be an effective way to improve adherence to treatment and medications (Baker, 2006; Wolf et al., 2007; Zhang, Terry, & McHorney, 2014). Health literacy refers to the knowledge, motivation, competency, understanding and appraisal of general health-related and healthcare information, by applying knowledge to the reading and understanding of medicine and nutrition labels, and understanding instructions provided by doctors (Sørensen et al., 2012). One meta-analysis identified consistent positive associations between higher health literacy levels and a greater ability to appropriately take and adhere to medications (Berkman et al., 2011). A second meta-analysisn found that patients with higher levels of health literacy were 14% better at adhering to their medications, compared to those with low health literacy, and that health literacy interventions effectively improved adherence to treatment and medications (Miller, 2016). Albeit both associations were considered weak (*r* = .14 and .16 respectively). Similarly, another meta-analysis identified that 14 out of 20 identified studies exploring the relationship between health literacy and medication adherence in chronic disease populations found positive associations between the two, with zero studies reporting a negative association (Neter & Brainin, 2019). However, two the of the discussed meta-analyses (Berkman et al., 2011; Neter & Brainin, 2019) report a low strength of evidence, mostly due to the research designs, and thus these associations may be biased.

Previous research has shown there is a relationship between health literacy and knowledge in chronic illness (Chajaee, Pirzadeh, Hasanzadeh, & Mostafavi, 2018; Yeh et al., 2018), suggesting that those with higher health literacy tend to also have higher levels of knowledge regarding the chronic illness they experience (Chajaee et al., 2018; Yeh et al., 2018). In a narrative review synthesizing the relationship between health literacy, knowledge and adherence to anticoagulants in cardiovascular disease there was an overall positive relationship between health literacy and knowledge (Cabellos-García et al., 2018). Participants with lower levels of health literacy and knowledge were also found to be less likely to adhere to their medications (Cabellos-García et al., 2018), and other studies have reported similar findings between health literacy, knowledge and adherence (Rolls, Obamiro, Chalmers, & Bereznicki, 2017). While there is some research examining knowledge and adherence to the pill (Hall et al., 2014), there is currently no literature which explores the influence and relationships between health literacy, knowledge and adherence to the oral contraceptive pill. Therefore, this study aimed to do so. Health literacy is often explored in specific illnesses (e.g. cardiovascular disease) or specific populations (e.g. Latinx) (Office of Disease Prevention and Health Promotion, 2020), but given that approximately 80% of women in the US report ever using the pill (Daniels & Abma, 2018), and the negative consequences of less than perfect adherence, it is important to understand the potential role that health literacy plays in this behavior to ensure women with inadequate health literacy are provided with the necessary skills to effectively adhere to the pill.

In addition, this study also sought to explore the interaction between health literacy and oral contraceptive pill knowledge in adherence to the pill. As general health knowledge and the application of this knowledge are key aspects of health literacy (Sørensen et al., 2012), the interaction between specific oral contraceptive pill knowledge and health literacy was considered to be an interesting avenue to explore. It may be that medication or disease-specific knowledge interacts with health literacy (and general health knowledge) to improve adherence. Knowledge in this study specifically refers to knowledge of the oral contraceptive pill and its risks, benefits, side effects, effectiveness, use and mechanisms of action (Hall et al., 2014; Hall et al., 2010a ). It was hypothesized that based on previous literature, there would be a positive and significant association between (i) health literacy and adherence, (ii) knowledge of the oral contraceptive pill and adherence, and (iii) health literacy and knowledge. Secondly, it was hypothesized that after controlling for age and education, both health literacy and knowledge would be directly associated with adherence to the oral contraceptive pill. Thirdly, it was hypothesized that knowledge would moderate the relationship between health literacy and adherence, such that there would be a significant positive association between health literacy and adherence but at high levels of knowledge.

## Methods

### Procedure

A cross-sectional design was used, and the study was approved by the University’s Human Research Ethics Committee (HRE2017–0173). Data were collected online through the crowdsourcing platform CloudResearch (cloudresearch.com), owned by Amazon Mechanical Turk, in early 2020. To be eligible for participation, participants were required to have female reproductive anatomy and be currently taking the oral contraceptive pill (either the combination pill or the progestogen-only pill). All eligible participants provided informed and once consent was provided, all participants completed measures of general health literacy, knowledge of the oral contraceptive pill, adherence to the oral contraceptive pill over the previous month and demographic questions both related to the oral contraceptive pill and general demographics. The online questionnaire was completed on Qualtrics using a device of the participants choice. The survey took no longer than 15 mins to complete and participants were reimbursed $2USD for their time.

### Measures

#### Health literacy

General health literacy was measured using the Health Literacy Skills Instrument – Short Form (HLSI-SF; Bann, McCormack, Berkman, & Squiers, 2012). This measure is a shortened 10-item version of the original 25-item Health Literacy Skills Instrument (McCormack et al., 2010). The short form contains five items that measure print literacy (reading, locating and understanding health information), two that measure numeracy (seeking and using quantitative information), two measuring oral literacy (listening effectively and understanding), and one item that requires the participants to locate health information through the internet. Items required participants to listen to short audio clips, watch videos or examine print documents such as pamphlets and hospital maps. For each item, there is only one correct answer, with correct answers summed together to create a final score out of 10. Final scores of 6 or below are considered ‘inadequate health literacy’ and scores equal to or greater than 7 are considered ‘adequate health literacy’ (Bann et al., 2012). The instrument showed acceptable reliability in our sample (α = .75).

#### Knowledge

Levels of oral contraceptive pill knowledge were measured using an established measure created by Hall, Westhoff, and Castaño (2013). The 41-item measure assesses the six main dimensions of oral contraceptive knowledge: mechanism of action (‘The pill has either a combination of estrogen and progestin or progestin-only’), effectiveness (‘The regular use of certain medications can reduce how well the pill works’), use (‘The pill is a daily hormonal contraceptive’), side-effects (‘The pill causes weight gain’), risks (‘You cannot get sexually transmitted infection while taking the pill’) and benefits (‘Does the pill make menstrual cramps better, worse or have no effect?’). The questions employed a range of formats such as true/false (10 items), multiple choice (15 items) and alternative choice (16 items). Each item only had one correct answer. All correct answers were summed to create a final score out of 41. The higher the score, the better knowledge the participant has of the oral contraceptive pill.

#### Adherence

Adherence to the oral contraceptive pill over the previous month was measured using the Medication Adherence Report Scale (MARS; Horne & Weinman, 1999), adapted to ask questions related to oral contraceptive pill adherence (Molloy et al., 2012). The measure contains five items asking participants about their adherence, or lack thereof, to the oral contraceptive pill. Each item was answered on a 5-point Likert scale from 1 = never to 5 = always (e.g. ‘I forgot to take my oral contraceptive pill’). All items were reverse coded for ease of interpretation, and scores were summed, with higher scores meaning greater adherence over the previous month. The MARS demonstrated reliability in our sample (α = .97).

#### Demographics

Participants were asked several demographic questions related to sexual activity, pregnancy, details of their oral contraceptive pill (name, length of time used, the reason for taking, current routine), experience obtaining the oral contraceptive pill, and any instructions they were provided when prescribed. General demographics such as age, gender, annual income, education and ethnicity were also asked.

### Data analysis

All data were analyzed using IBM SPSS Statistics Version 26 (IBM Corp, 2019). Cases that selected they did not take the pill (*n *= 80) or did not provide informed consent (*n* = 3) were removed before a missing values analysis being conducted. A missing values analysis was conducted on the remaining cases and Little's MCAR test showed that data were not missing completely at random, *χ*^2^(2525) = 2789.62, *p* <.001. A total of 49 participants missed one or more items in the knowledge measure, with 24 of these missing 5% or more. A large majority of the missing data points were in a single subscale of the knowledge measure, with each item missing between 5.2–6.7% of its data. As listwise deletion of cases would be inappropriate, multiple imputation with 5 imputations using fully conditional specification was conducted. Subsequent analysis was conducted on pooled statistics.

Descriptive statistics and Pearson’s correlation coefficients were conducted on the unstandardized variables. Variables were standardized before conducting the hierarchical multiple regression analysis to reduce multicollinearity (Aiken & West, 1991). A Mann–Whitney *U* test was conducted to examine any differences in adherence and health literacy in those who missed any items on the knowledge measure and those that did not. Results showed significant differences in adherence to the pill between those missing at least one item and those not missing any, *U* = 2234.00, *z* = −3.91, *p* <.001, such that those not missing any items reported greater adherence. Similarly, the results indicated significant differences in health literacy between those that missed an item and those that did not, *U* = 2129.50, *z* = −4.18, *p* <.001. Those who missed no items exhibited better health literacy.

Education level was not significantly correlated with any of the key variables, so the decision was made to remove it from the regression analysis. However, age, ethnicity and years taking the pill were all significantly correlated with either health literacy, knowledge or adherence and were subsequently controlled for in the regression. At step one age, ethnicity and years taking the pill were entered and controlled for. At step two health literacy was entered, at step three oral contraceptive pill knowledge was entered, and at step four, the interaction between health literacy and knowledge was entered.

## Results

### Participants

After the removal of cases, a total of 193 participants remained in the sample (*M*_age_ = 32.63 years, *SD* = 5.98). All participants identified as female, 60.6% identified as Caucasian and 26.4% identified as African American, 79% of participants had at least a Bachelor’s degree and 86% earnt USD 75,000 or less annually. Concerning the oral contraceptive pill, 86% of participants reported taking the combination pill. Time taking the pill ranged from 1 month up to 37 years, with a mean of 3.99 years. A majority of the sample was taking the pill for contraception (53.6%), however, other reasons such as to regulate periods (18.2%), to help with acne or skin problems (10.9%) or to reduce the effects of menstrual cramps or endometriosis (9.8%) were also common. With regards to sexual activity and pregnancy demographics, 85.5% of the sample reported being sexually active, 45.6% reported ever being pregnant and 48.19% had children. Participants’ health literacy was largely inadequate, with *N* = 116 exhibiting inadequate health literacy (≤ 6 out of 10 correct responses) and *N =* 77 exhibiting adequate health literacy (>7 out of 10 correct responses). In total, participants’ knowledge of the pill was on the midpoint of the scale (*M* = 20.38 out of 41, *SD* = 5.64) and adherence in the sample was good (*M* = 18.70 out of 25, *SD* = 6.85).

### Bivariate analyses

A Chi-square test of contingencies indicated a significant difference in health literacy levels and ethnicity, *χ*^2^(3, *N* = 191) = 20.07, *p *< .001, such that those who identified as African American were more likely to have inadequate health literacy (*n* = 42) compared to adequate health literacy (*n* = 7). Results were similar for those that defied as Latinx, with *n *= 8 having inadequate health literacy, while *n* = 4 had adequate health literacy. In comparison, there was a relatively even number of people who identified as Caucasian having inadequate (*n* = 57) or adequate (*n* = 60) health literacy in this sample.

An independent samples *t*-test was computed to examine any differences in the (i) mean knowledge scores between the two health literacy groups, and (ii) adherence levels in those with inadequate and adequate health literacy. There was a significant difference between the two health literacy groups and mean knowledge score. Those with adequate health literacy (*M* = 23.26, *SD* = 5.38) achieved a mean of 4.78 more correct knowledge questions, 95% CI [3.30, 6.28] compared to those with inadequate health literacy (*M* = 18.47, *SD* = 4.97), *t*(189) = 6.30, *p* <.001, Hedges’ *g* = .83. Similarly, there was a significant difference in adherence between the two health literacy groups, with those with adequate health literacy (*M* = 24.23, *SD* = 2.13) reporting better mean adherence to the oral contraceptive pill by 9.19 points, 95% CI [7.91, 10.47] than those with inadequate health literacy (*M* = 15.03, 6.43), *t*(146.63) = 14.07, *p* <.001, Hedges’ *g* = 1.86.

Table 1 shows the means, standard deviations, and correlations between the main demographic variables, health literacy, knowledge and adherence. Age was significantly weakly positively associated with number of years taking the pill (*r* = .29, *p* <.001) and knowledge (*r* = .15, *p* = .040). Education level was not significantly associated with the level of health literacy, knowledge or adherence. Health literacy was significantly associated with number of years taking the pill (*r* = .27, *p* <.001), knowledge (*r* = .42, *p* <.001) and adherence (*r* = .76, *p* <.001). Knowledge was moderately correlated with number of years taking the pill (*r* = .24, *p* = 001) and adherence (*r* = .40, *p* <.001).

**Table 1.** Means, Standard Deviations and Correlations between Key Demographic Variables, Health Literacy, Knowledge and Adherence

	M	SD	1	2	3	4	5	6
1.Age	32.63	5.99	–	−.01	.29**	.10	.15*	.07
2.Education	–	–		–	.01	−.02	−.02	−.03
3.Number years taking pill	3.99	5.58			–	.27**	.24**	.33**
4.Health Literacy	5.26	2.42				–	.42**	.76**
5. Knowledge	20.38	5.64					–	.40**
6. Adherence	18.70	6.85						–

Note*.* M = mean, SD = standard deviation.

*p < .05, **p < .01.

### Hierarchical multiple regression analysis – predicting adherence

In step one of the analysis, age, ethnicity and number of years taking the pill were entered and accounted for a significant 17.4% of the variance in adherence to the oral contraceptive pill, *R*^2^ = .17, *F*(3, 179) = 12.53, *p* <.001. In step two health literacy was entered and accounted for a significant 41.8% of the variance in adherence to the oral contraceptive pill, Δ*R*^2^ = .42, Δ*F*(1, 178) = 182.00, *p*<.001. In step three knowledge was added to the regression and accounted for no additional variance, Δ*R*^2^ = .006, Δ*F*(1, 177) = 2.68, *p* = .103. In step four the interaction term between health literacy and knowledge was added to the model and accounted for no additional variance, Δ*R*^2^ = .001, Δ*F*(1, 176) = 0.41, *p* = .521. In combination, the three variables accounted for a total 59.8% of the variance in adherence to the pill, *R*^2^ = .60, *F*(6, 176) = 834.61, *p*<.001. The only significant predictors in the final model were the number of years taking the pill (*p* = .018) and health literacy (*p*<.001). See Table 2 for the regression coefficients.

**Table 2.** Unstandardized (*B*) and Standardized Regression Coefficients (*β*), and Squared Semi-Partial Correlations (*sr*^2^) for Each Predictor and Step in the Hierarchical Multiple Regression.

	Variable	*B* [95% CI]	β	*sr*^2^	*p*	*R*^2^	Δ*R*^2^	*F*	Δ*F* (*df*1, *df*2)
Step 1					.**000**	.**174**	.**174**	**12**.**52**	**12.52** (***3,179*)**
	Age	−.04 [−.20, .11]	−.04	.00	.583				
	**Ethnicity**	1.09 [.53, 1.65]**	.27	.07	.000				
	**Numbers of years taking pill**	.33 [.16, .51]**	.28	.07	.000				
Step 2					.**000**	.**591**	.**418**	**64**.**40**	**182.00** (***1, 178*)**
	Age	−.05 [−.16, .06]	−.04	.00	.389				
	Ethnicity	.12 [−.30, .55]	.03	.00	.562				
	**Number of years taking pill**	.17 [.04, .29]*	.14	.02	.009				
	**Health literacy**	4.90 [4.18, 5.51]**	.71	.42	.000				
Step 3					.**000**	.**597**	.**006**	**52**.**55**	**2.68** (***1, 177*)**
	Age	−.06 [−.17, .05]	−.05	.00	.314				
	Ethnicity	.09 [−.33, 51]	.02	.00	.665				
	**Number of years taking pill**	.15 [.03, .28]*	.13	.01	.015				
	**Health literacy**	4.70 [3.95, 5.44]**	.69	.35	.000				
	Knowledge	.60 [−.12, 1.30]	.09	.00	.103				
Step 4					.**000**	.**598**	.**001**	**43**.**71**	**0.41** (***1, 176*)**
	Age	−.06 [−.17, .05]	−.05	.00	.317				
	Ethnicity	.08 [−.34, .50]	.20	.00	.698				
	**Number of years taking pill**	.15 [.03, .28]*	.13	.01	.018				
	**Health literacy**	4.73 [3.97, 5.50]**	.69	.34	.000				
	Knowledge	.51 [−.23, 1.27]	.08	.00	.174				
	Health literacy X knowledge	.26 [−.55, 1.10]	.03	.00	.521				

Note*.* CI = confidence intervals.

*p < .05, **p < .01.

## Discussion

In this study, we explored the associations between health literacy, knowledge and adherence to the oral contraceptive pill. The primary findings of this study suggest that health literacy has the strongest association with adherence to the oral contraceptive pill and explains the most variance in adherence. However, the length of time taking the pill is also important. The findings also suggest that adherence to the oral contraceptive pill is relatively high, however improving health literacy skills may be a way to improve adherence to the pill and subsequently improve its effectiveness at preventing pregnancy and improving its non-contraceptive benefits.

We found significant positive associations between health literacy and adherence, knowledge and adherence, and knowledge and health literacy. All three correlations were positive, however, the association between health literacy and adherence to the pill was the strongest and the remaining two associations were moderate. This association is consistent with previous research which also found a significant association between health literacy and medication adherence across a range of chronic diseases (Mayo-Gamble & Mouton, 2018; Miller, 2016; Osborn et al., 2011; Zhang et al., 2014). We have now also demonstrated that this is true for taking the pill.

We found a significant positive association between oral contraceptive knowledge and adherence, which supports previous research (Hall et al., 2014; Tomaszewski et al., 2017), however, this became non-significant when health literacy was included, perhaps due to shared variance. This suggests knowledge, along with health literacy, is related to adherence such that those with higher knowledge of the pill tend to also have better adherence to the pill. Women who attend pill related check-ups or to receive repeat prescriptions could be asked a few questions related to the oral contraceptive pill, such as its use, benefits or mechanisms of action, to get an understanding of their knowledge related to the pill. If their knowledge is low, educational resources or leaflets should be provided to educate the individual as well as to hopefully improve their overall health knowledge, which is an aspect of health literacy (Sørensen et al., 2012). Similarly, when women are first prescribed the pill they should receive a simple educational leaflet from the health professional (Little, Griffin, Kelly, Dickson, & Sadler, 1998) to ensure they are knowledgeable before they begin taking the pill, rather than just the leaflet in the pillbox which tends to have poor readability and is read by a minority of people (Koo, Krass, Aslani, Guzmàn, & Le Duff, 2003; Raynor & Knapp, 2000). However, providing educational leaflets and asking health-related questions may be ineffective in improving knowledge if the patient has inadequate health literacy. This is further supported by our finding of a significant positive association between health literacy and oral contraceptive pill knowledge. This relationship has previously also been identified in chronic illness understanding (Chajaee et al., 2018; Gazmararian, Williams, Peel, & Baker, 2003; Van Der Heide et al., 2014; Yeh et al., 2018), such that patients with low health literacy also have less of an understanding of their chronic illness. Although associations are unable to show causality, knowledge is a component of health literacy (Sørensen et al., 2012) and therefore our findings may demonstrate that perhaps general health literacy skills need to be targeted and improved first, before patients are provided with specific oral contraceptive pill knowledge.

Health literacy was the most significant predictor of adherence to the oral contraceptive pill with neither knowledge nor the interaction between health literacy and knowledge significant predictors. This finding is not uncommon in the literature for other chronic diseases (Lee, Yu, You, & Son, 2017; Murray et al., 2004). It may be that knowledge of the oral contraceptive pill is important for understanding *how* to adhere to the pill correctly but is not enough to predict adherence. It may be that additional determinants of adherence to the pill, such as perceived behavioral control/self-efficacy (Ajzen, 1991; Molloy et al., 2012) or beliefs related to medication (Horne & Weinman, 1999), need to be explored further. For instance, women who view taking the pill as ‘easy’ and ‘simple’ may have greater adherence and similarly, those that view taking the pill more positively may also exhibit greater adherence. Perceived behavioral control and self-efficacy have previously been identified as being significant predictors in adherence to the pill, although only in one study (Molloy et al., 2012). Similarly, in an older study, negative beliefs about the oral contraceptive pill negatively influenced both intentions to use and adherence to the pill (Moore, Adler, & Kegeles, 1996). Both psychosocial variables should be further investigated to see if they are influential above and beyond health literacy.

Similarly, due to the moderate association between health literacy and knowledge, it is possible that the basic knowledge of the oral contraceptive pill (e.g. understanding how to read the medication label and instructions) was accounted for in the health literacy measure. However, further research is needed to explore the relationship between health literacy and knowledge of the pill. These findings suggest that family planning clinics should consider assessing the patient's level of health literacy, perhaps using an assessment tool designed for use in clinical settings, such as the Newest Vital Sign (NVS; Osborn et al., 2007; Weiss et al., 2005). By doing so, they can grasp a deeper understanding of the patients understanding of health-related information and provide additional resources, or spend more time educating the patient on understanding the labels and instructions associated with their oral contraceptive pill.

Health professionals need to spend more time and effort with patients with poorer health literacy to ensure they fully comprehend what is required of them, with clinicians providing strategies such as patient-centred communication (e.g. ‘what do you already know about the pill?’), being clear about health topics (e.g. using plain language and attempting to match the patient's vocabulary) and confirming understanding with the patient before allowing them to leave (Kountz, 2015; Sudore & Schillinger, 2009). If this is not viable due to time restraints, it may be worthwhile referring patients to lay health educators in their community. Lay health educators can provide effective communication and health support across all literacy levels (Auger & Verbiest, 2007) if the clinician does not have the appropriate skills to do so. Also, health professionals need to ensure they are creating an empowering environment such as making forms clear and easy to read, as well as ensuring they are up to date with their medical and health education accreditations so they are best able to assist their patients (Sudore & Schillinger, 2009). Similarly, in interventions aimed at improving health literacy it may be that targeting the individual components of health literacy (e.g. reading) will be more useful in yielding significant improvements in health literacy, rather than trying to target the entire concept at the one time. Small changes in general literacy can result in a better overall application of the skills to specific health situations (Nutbeam, McGill, & Premkumar^2018^, ).

It is also important to acknowledge that length of time taking the pill was also predictive of adherence. Interpreted in conjunction with the significant positive association between the number of years taking the pill and adherence, it suggests that females who have been taking the pill for longer are better at adhering. This relationship should be explored further to see whether the behavior has perhaps become routinized or even become automatic such that it is now a habit. Habit has previously shown to be associated with better adherence to the oral contraceptive pill (Murphy, Eustace, Sarma, & Molloy, 2018), but only in a single study. The role of habit in understanding adherence to the oral contraceptive pill could be further explored within theories of behavior that incorporate habit, such as temporal self-regulation theory (Hall & Fong, 2007), as other psychological variables may be more predictive of adherence than habit. Temporal self-regulation theory has previously been shown to be useful in predicting general medication adherence (Liddelow et al., 2020a).

Finally, approximately 60% of our sample exhibited inadequate health literacy, which is consistent with government statistics (Australian Commission on Safety and Quality in Health Care, 2014). Our findings also showed there to be significant disparities in the level of health literacy (inadequate and adequate) based on ethnicity. Of the participants that identified as African American, 86% exhibited inadequate health literacy. Further, those with inadequate health literacy were less knowledgeable about the oral contraceptive pill and reported worse adherence to the pill. These findings provide insight into the groups where improvements in health literacy are needed, and also reflects the possible systemic inequalities in modern society.

### Strengths and limitations

To the best of our knowledge, this is the first study to examine the relationships between health literacy, knowledge, and adherence to the oral contraceptive pill. A strength of the study is the wide demographic surveyed. This increases the generalizability of the findings and associated implications for various populations outside of the current sample. There are however limitations to this study, including the use of a cross-sectional design, which precludes any conclusions regarding the direction of the associations as well as causality. In addition, crowdsourcing platforms also present a range of their own limitations such as the presence of bots and the use of duplicate IP addresses (Cheung, Burns, Sinclair, & Sliter, 2017). However, to reduce the occurrence of these limitations, specific settings and options were selected on CloudResearch to ensure the validity and quality of our data. Furthermore, the alternative choice items in the knowledge measure (Hall et al., 2014) seemed to present issues with missing data. Each of the 16 items presented in this format was missing a considerable amount of data compared to the true/false and multiple-choice formatted questions. Similarly, those with lower health literacy levels were more likely to miss items on this measure indicating the items or the way it was presented was too complex for a sample largely comprised of individuals with inadequate health literacy. Future research may consider removing these alternative choice questions to ensure the simplicity of the measure for participants. The final limitation of the current study is the measurement of adherence to the pill. Direct measures of adherence to the pill, such as through serum or urine samples (Hall et al., 2010b), would provide better estimations of adherence, however, are more expensive and difficult to administer, particularly in an online survey and therefore self-report was the preferred chosen measure of adherence despite limitations associated with self-report measures. Our measure of adherence was skewed towards greater adherence, however still presented a range of adherence, from the lowest possible score to the highest.

## Conclusion

The present study explored the associations between health literacy, knowledge of the oral contraceptive pill and adherence, as well as the predictive ability of these two variables in predicting adherence to the oral contraceptive pill. Results showed that health literacy and knowledge are both significantly positively associated with adherence; however, health literacy was the strongest predictor of adherence to the pill in multivariate analyses. As this is the first study to explore these relationships, future research is needed to replicate the findings of this study. However, the findings suggest that health professionals should consider assessing patient’s health literacy skills when prescribing the oral contraceptive pill to ensure patients fully understand how/what to do to successfully adhere and ensure the pills’ effectiveness. Those that exhibit lower levels of health literacy should be provided with additional resources related to taking the pill correctly. Subsequently, it is hoped that by increasing health literacy levels, adherence to the oral contraceptive pill will be improved.
